# Forecasting crude oil futures price with energy uncertainty: Evidence from machine learning methods

**DOI:** 10.1371/journal.pone.0341496

**Published:** 2026-02-05

**Authors:** Xiaolu Wei

**Affiliations:** Business School, Hubei University, Wuhan, China; University of Oklahoma, UNITED STATES OF AMERICA

## Abstract

Energy related uncertainty has significant influence on crude oil market. To explore the influence, this paper investigates the predictive ability of the Energy-Related Uncertainty Index (EUI), over and above standard macroeconomic predictors, in forecasting crude oil prices using an array of machine learning methods. We find that EUI has a significant impact on crude oil prices. Moreover, machine learning methods combined with EUI performed better than the linear regression method due to a lower rate of prediction errors. Among these methods, the Random Forest (RF) model with EUI performs better in the short term, while the Attention-enhanced Long Short-Term Memory (Attention-LSTM) model with EUI has more substantial predictive power in the long term. These empirical results pass a series of robustness tests. Our findings have important implications for both regulators and investors in the crude oil market.

## Introduction

As one of the most important commodities in the world, crude oil is the foundation of modern industry and the global economy. Over the last few decades, researchers have found that crude oil price affect many goods and services that have a direct impact on the economy [1,2]. However, to accurately predict crude oil prices remains a significant challenge both theoretically and practically.

Since the introduction of economic policy uncertainty (EPU) by Baker et al. [3], the impact of uncertainty on the crude oil market and its application to forecasting crude oil price has gained considerable attention from scholars and policymakers. Until now, a large number of studies have been conducted to investigate the linkage between uncertainty and the crude oil market using indices related to uncertainty. For example, Li et al. [4] use multiple uncertainty indicators to forecast crude oil volatility and found that the U.S. petroleum market equity market volatility tracker index (PMEMV) performs better in forecasting short-term crude oil volatility, while the geopolitical risk index (GPR) has better predictive power for short-term crude oil volatility. Dai et al. [5] investigate whether the global economic policy uncertainty (GEPU) and the change of GEPU (GEPU) have different impacts on crude oil futures volatility under the single-factor model and two-factor model. The findings show that the one-factor model with GEPU or GEPU is consistently effective in predicting the volatility of crude oil futures, while the two-factor model with GEPU change has a much stronger forecasting ability than that with GEPU. Nonejad [6] evaluates whether the geopolitical risk (GPR) index could improve the prediction accuracy of crude oil price volatility and what forms of nonlinearity provide the largest degree of forecast accuracy gains. They find that GPR increases over the recent maximums offer the highest degree of forecast accuracy gains, while GPR decreases below recent minimums do not deliver such gains.

While previous studies show that uncertainty can not only affect crude oil markets but also predict crude oil prices, few studies have examined the predictive power of energy-related uncertainty on crude oil prices. Moreover, existing studies mainly explore the predictive ability of uncertainty on crude oil prices through traditional methods such as linear regression model (LR) [7,8], autoregressive model (AR) [9,10], and generalized autoregressive conditional heteroskedasticity model (GARCH) [11–13]. These methods are too dependent on the quality of the data, which needs to be stationary [14,15]. Furthermore, these methods usually ignore the possible non-linear relationship between uncertainty and crude oil prices, which carries the risk of underfitting [16,17]. To solve these problems, this study examines the predictive ability of the energy-related uncertainty index (EUI) on crude oil prices based on machine learning methods. Compared to traditional methods, machine learning methods are effective not only in solving the problem of data pre-processing but also in capturing the non-linear relationship between uncertainty and crude oil prices.

The main purpose of this study is to answer two vital questions: 1. Will the inclusion of EUI improve the accuracy of crude oil price prediction? 2. Which model will most effectively predict crude oil price? Following this logic, we first collect data on the EUI and other factors that may influence crude oil price. Then, we employ the Least Absolute Shrinkage and Selection Operator (LASSO) regression test to select other variables with significant predictive power. Finally, we use six types of models, including one traditional model (Linear Regression model (LR)), one machine learning method (support vector regression (SVR)), two deep learning methods (Long Short-Term Memory (LSTM), Attention-enhanced Long Short-Term Memory (Attention-LSTM)), and two tree-based techniques (Random Forest (RF), and Extreme Gradient Boosting (XGBoost)), to predict crude oil price. The LR model is employed as a simple, interpretable baseline. The selection of other five models is motivated by their ability to capture the diverse and complex characteristics of crude oil price movements. Specifically, LSTM and Attention-LSTM are adept at learning from sequential data. SVR, RF, XGBoost have strong capability in capturing non-linear patterns and complex interactions among features. The results indicate that the RF model that incorporates EUI excels in the short-term prediction by robustly handling noisy, non-linear feature interactions dominant in immediate price movements. Conversely, the Attention-LSTM model combined with EUI dominates long-term forecasts, as its sequential learning and attention mechanism are better suited to distill the underlying long-range dependencies that govern crude oil prices trends.

This study makes three primary contributions to the energy finance literature. First, we are among the first to integrate LASSO feature selection with the Energy Uncertainty Index (EUI) for crude oil forecasting, thereby providing a novel and tailored framework to assess uncertainty’s impact on the oil market. Second, we identify the most relevant predictors from a broad set of variables, which not only enhances forecasting precision but also improves model interpretability and guards against overfitting. Finally, we deploy a diverse suite of machine learning algorithms to separately forecast short-term and long-term crude oil prices, enabling a comprehensive comparison of their predictive performance across different time horizons. From a practical standpoint, our findings offer actionable insights for investors and policy regulators in managing oil price risks and formulating data-driven strategies.

The remainder of this paper is organized as follows. Section 2 describes the data used in this study. Section 3 describes the methodologies utilized. Section 4 shows the empirical results. Section 5 presents various robustness tests. Section 6 provides our conclusions.

## Data

### Crude oil futures prices

**Fig 1:**
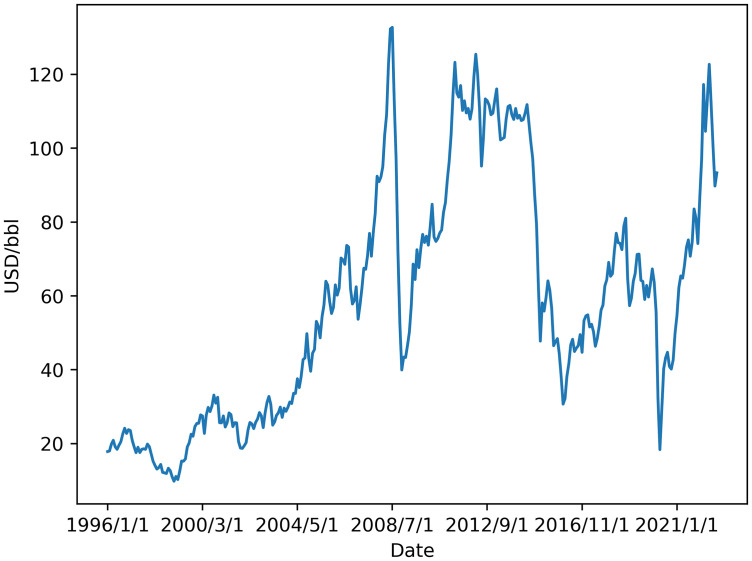
The crude oil price of Brent.

We focus on Brent oil futures, a key global benchmark, as it represents the international pricing standard for crude oil. The monthly close price for Brent is collected from the U.S. Energy Information Administration (EIA). The sample period spans from January 1996 to October 2022, encompassing major events such as the Iraq War (2003), the global financial crisis (2008–2009), the international oil price crash (2014), and the COVID-19 pandemic (2020). As illustrated in Fig 1, these events induced substantial price volatility. This underscores the practical need for market participants and policymakers to forecast the future price levels for investment and planning, rather than relying solely on period-to-period returns.

Consequently, our study predicts crude oil price levels directly. Although price levels may exhibit non-stationarity, the machine learning methods we employ are adept at learning from such data without relying on the strict stationarity assumptions required by traditional econometric models. Their ability to capture complex, persistent patterns is a key advantage in this context. Additionally, we predict returns, as detailed in the Robustness Tests section to address any concerns regarding potential non-stationarity.

### EUI and other predictors

In this paper, we use two kinds of global EUI introduced by Dang et al. [18] to investigate the impact of energy uncertainty on crude oil prices, including the equal-weighted EUI (EUI_equally) and the GDP-weighted EUI (EUI_GDP). The data of EUI is available from the official website of EPU (http://www.policyuncertainty.com/energy_uncertainty.html), where the sample range is also from January 1996 to October 2022.

Furthermore, many other factors affect the price of crude oil futures. Therefore, we must consider extensive aspects in our prediction model. Based on previous studies, we also include 24 potential factors in five categories as predictors, as shown in Table 1. Concerning the bond market, we select five benchmark interest rates—the federal funds rate, treasury bill rate, London Interbank Offered Rate, default yield spread, and government long-term bond yield. The selection of these specific benchmarks is justified by their documented empirical significance in previous research as key drivers of crude oil prices [19–22]. For the stock market, we choose three factors: the S&P500 index, the Dow Jones Industrial Average, and stock variance. This aligns with Xu et al. [19], and Welch and Mensi [23]. We choose the S&P GSCI Non-Energy index and CRB Rind index, for the futures market following Xu et al. [19]. Concerning the economic state, we choose nine factors that are commonly used to predict crude oil price: unemployment rate, capacity utilization, the Chicago Fed’s national activity index, inflation rate, money supply, industrial production index, US dollar index, MSCI World Index, ISM Manufacturing Index [22]. Additionally, Zhang et al. [21], and Dai and Kang [22] have capitalized on crude oil production, crude oil import, and crude oil stock on predicting crude oil price. We thus select five factors to represent the impact of the related crude oil market, including growth of global crude oil production, growth of US crude oil production, growth of US crude oil stock, growth of US crude oil import, and growth of global crude oil import.

**Table 1:** Potential factors affecting crude oil futures price.

Factor Category	Factors	Abbreviations
Bond market	Federal Funds Rate	DFF
	Treasury bill rate	TBL
	The London Interbank Offered Rate	LIBOR
	Default yield spread	DFY
	Government long-term bond yield	LTY
Stock market	S&P500 index	S&P500
	Dow Jones Industrial Average	DJIA
	Stock variance	SVAR
Futures market	S&P GSCI Non-Energy index	GSCI-NE
	CRB Rind index	CRB
Economic state	Unemployment rate	UR
	Capacity utilization	CU
	The Chicago Fed’s national activity index	CFNAI
	Inflation rate	INFL
	Money supply	MS
	Industrial production index	IPI
	US dollar index	DOLLAR
	MSCI World Index	MSCI
	ISM Manufacturing Index	ISM
Related crude oil market	Growth of global crude oil production	GCOP
	Growth of US crude oil production	USCOP
	Growth of US crude oil stock	USCOS
	Growth of US crude oil import	USCOI
	Growth of global crude oil import	GCOI

Finally, the collected data is normalized and divided chronologically into three parts to prevent look-ahead bias. The first part is the training set (60% of the sample), which is used for preprocessing, feature selection and model training. The second part is the validation set (20% of the sample), which is used to tune hyper parameters through grid search method. The third part is the testing set (20% of the sample), which is used to evaluate the performance of the prediction model. The complete dataset supporting the findings of this study is available in S1 File.

## Methodology

### Feature selection methods

Feature selection plays a crucial role in the field of machine learning by selecting the most informative features from raw data, improving model performance, reducing overfitting, and speeding up model training and prediction. Feature selection is important in large datasets and high-dimensional data, as unnecessary features increase computational complexity and introduce redundant information. The overfitting phenomenon may occur when employing a prediction model, especially when some variables are strongly correlated. Therefore, we adopted LASSO regression [24] to filter the variables. Lasso is a linear regression method that adopts L1-regularization. The use of L1-regularization will make the weight of some learned features 0, to achieve the purpose of sparsity and feature selection.

The LASSO estimate is defined by the equation shown below:


$${\hat{\beta }}^{\text{lasso}}=\text{argmi}{\text{n}}_{\beta }\left\{\frac{\text{1}}{\text{2N}}{\sum_{\text{i}=\text{0}}^{\text{N}}{(\text{y}}_{\text{i}}-\beta_{\text{0}}-\sum_{\text{j}=\text{1}}^{\text{p}}{\text{x}}_{\text{ij}}\beta_{\text{j}})}^{\text{2}}+\lambda \sum_{\text{j}=\text{1}}^{\text{p}}\left|\beta_{\text{j}}\right|\right\}$$ (1)

Where N is the total number of observations, λ represents a nonnegative regularization parameter corresponding to one value of Lambda, ${\text{y}}_{\text{i}}$ is the dependent variable, p is the number of independent variables ${\text{x}}_{\text{i}}={\left({\text{x}}_{\text{i1}},\ldots ,{\text{x}}_{\text{ip}}\right)}^{\text{T}}$, $\beta_{\text{0}}$ symbolizes the intercept, and $\beta_{\text{j}}$ stands for the other parameters.

The loss function expression of LASSO is:


$$\text{J}(\theta )=\frac{\text{1}}{\text{2N}}{\left({\text{X}}_{\theta }-\text{Y}\right)}^{\text{T}}\left({\text{X}}_{\theta }-\text{Y}\right)+\alpha {\left\|\theta \right\|}_{\text{1}}$$ (2)

where α is the constant coefficient and needs to be tuned, ${\left\|\theta \right\|}_{\text{1}}$ is the L1 norm.

LASSO is characterized by variable selection and regularization while fitting the generalized linear regression model. Therefore, whether the target dependent variable is continuous, binary or multivariate discrete, it can be modeled and predicted. LASSO can solve problems with multicollinearity, allowing automatic feature selection for high-dimensional data sets. It can improve the generalization ability of the model and avoid overfitting. Moreover, LASSO can provide the sparsity of the coefficients, making the model more explanatory.

### Predictive models

#### Long short term memory model (LSTM).

RNNs are powerful and well suited for processing sequences of inputs. These networks have a certain persistence, which enables information to be passed from one time step to the next. However, their weakness is that they are unable to capture long-term historical information because they cannot store data for an extended period. Moreover, in the reverse model training process with long sequences, RNN will cause gradient explosion or gradient disappearance.

To overcome this shortcoming of the model, the long and short term memory model was proposed by Hochreiter S and Schmidhuber J [25]. It has four gated units that can adaptively regulate the information flow inside the unit. Unlike previous neural networks such as ANN, LSTM can master intense learning tasks that require long-term memory of events.

The specific process is clarified in the following equations:


$${\text{f}}_{\text{t}}=\sigma \left({\text{W}}_{\text{fx}}{\text{x}}_{\text{t}}+{\text{W}}_{\text{fh}}{\text{h}}_{\text{t}-\text{1}}+{\text{b}}_{\text{f}}\right)$$ (3)

$${\text{i}}_{\text{t}}=\sigma \left({\text{W}}_{\text{ix}}{\text{x}}_{\text{t}}+{\text{W}}_{\text{ih}}{\text{h}}_{\text{t}-\text{1}}+{\text{b}}_{\text{i}}\right)$$ (4)

$${\text{o}}_{\text{t}}=\sigma \left({\text{W}}_{\text{ox}}{\text{x}}_{\text{t}}+{\text{W}}_{\text{oh}}{\text{h}}_{\text{t}-\text{1}}+{\text{b}}_{\text{o}}\right)$$ (5)

$${\text{c}}_{\text{t}}={\text{f}}_{\text{t}}⊙{\text{c}}_{\text{t}-\text{1}}+{\text{i}}_{\text{t}}⊙\text{tanh}\left({\text{W}}_{\text{cx}}{\text{x}}_{\text{t}}+{\text{W}}_{\text{ch}}{\text{h}}_{\text{t}-\text{1}}+{\text{b}}_{\text{c}}\right)$$ (6)

Where ${\text{f}}_{\text{t}}$, ${\text{i}}_{\text{t}}$, and ${\text{o}}_{\text{t}}$ represent the forget gate, input gate, and output gate for the sigmoid method, respectively. At time t, ${\text{x}}_{\text{t}}$ represents the input vector and ${\text{h}}_{\text{t}}$ signifies the hidden state vector, which is also known as the output vector of the LSTM unit that employs the elementwise product method signified as ⊙. W and b are weight matrices and bias parameters that must be learned during training, a process in which an LSTM neuron learns the hidden state (${\text{h}}_{\text{t}-\text{1}}$) of the previous neuron, and the current input it. After passing through the gate unit, the neural network obtains the output ${\text{o}}_{\text{t}}$ and passes the hidden state ${\text{h}}_{\text{t}}$ on to the next unit. There is a mechanism in the unit for determining the reset and update gates to control the quantity of information that will be ignored or used.

In this paper, the LSTM model is implemented and tuned with the following hyperparameters: the number of LSTM layers: [1, 2], the number of hidden units per layer: [32, 64, 128], the dropout rate: [0.1, 0.2, 0.3], the optimizer: [‘Adam’, ‘RMSprop’], the learning rate: [0.001, 0.005], and the batch size: [16, 32, 64]. We employ early stopping with a patience of 15 epochs to determine the training epochs. The final model architecture that yields the best performance consists of two stacked LSTM layers with 64 and 32 hidden units respectively, followed by a fully connected output layer. We incorporate dropout layers with a rate of 0.2 after each LSTM layer. The model is trained using the Adam optimizer with a learning rate of 0.001 and a batch size of 32. The typical number of training epochs ranges between 80 and 120.

#### Attention-enhanced long short-term memory (Attention-LSTM).

Attention-LSTM model is a hybrid neural architecture that merges the sequential modeling capabilities of LSTMs with adaptive attention mechanisms to overcome limitations in processing long-term dependencies. Originating from the attention framework proposed by Bahdanau et al. [26] for neural machine translation, Attention-LSTM computes attention scores to dynamically weight hidden states, addressing the “information bottleneck” in standard LSTMs. In vanilla LSTM, the hidden state ${\text{h}}_{\text{t}}$ at time t evolves through gating mechanisms:


$${\text{f}}_{\text{t}}=\sigma \left({\text{W}}_{\text{f}}[{\text{h}}_{\text{t}-\text{1}}{,\text{x}}_{\text{t}}]+{\text{b}}_{\text{f}}\right)$$ (7)

$${\text{i}}_{\text{t}}=\sigma \left({\text{W}}_{\text{i}}[{\text{h}}_{\text{t}-\text{1}}{,\text{x}}_{\text{t}}]+{\text{b}}_{\text{i}}\right)$$ (8)

$${\text{o}}_{\text{t}}=\sigma \left({\text{W}}_{\text{o}}[{\text{h}}_{\text{t}-\text{1}}{,\text{x}}_{\text{t}}]+{\text{b}}_{\text{o}}\right)$$ (9)

$${\tilde{\text{c}}}_{\text{t}}=\text{tanh}\left({\text{W}}_{\text{c}}[{\text{h}}_{\text{t}-\text{1}}{,\text{x}}_{\text{t}}]+{\text{b}}_{\text{c}}\right)$$ (10)

$${\text{c}}_{\text{t}}={\text{f}}_{\text{t}}⊙{\text{c}}_{\text{t}-\text{1}}+{\text{i}}_{\text{t}}⊙{\tilde{\text{c}}}_{\text{t}}$$ (11)

$${\text{h}}_{\text{t}}={\text{o}}_{\text{t}}⊙\text{tanh}({\text{c}}_{\text{t}})$$ (12)

where σ denotes the sigmoid function and ⊙ is element-wise multiplication. However, this structure struggles to prioritize critical time steps in long sequences. Attention-LSTM addresses this by introducing a context vector ${\text{context}}_{\text{t}}$, calculated as a weighted sum of encoder hidden states ${\text{h}}_{\text{j}}$:


$$\begin{array}{c} {\text{context}}_{\text{t}}=\sum {\mathrm{\backslash nolimits}}_{\text{j}=\text{1}}^{\text{T}}\alpha_{\text{tj}}{\text{h}}_{\text{j}} \end{array}$$ (13)

where the attention weights $\alpha_{\text{tj}}$ are derived from a softmax-normalized alignment score:


$$\alpha_{\text{tj}}=\frac{\text{exp}(\text{score}({\text{h}}_{\text{t}}^{\text{dec}},{\text{h}}_{\text{j}}^{\text{enc}}))}{\sum_{\text{k}=\text{1}}^{\text{T}}\text{exp}(\text{score}({\text{h}}_{\text{t}}^{\text{dec}},{\text{h}}_{\text{k}}^{\text{enc}}))}$$ (14)

The alignment score function, often implemented as additive $\left({\text{v}}^{\text{T}}\text{tanh}\left({\text{W}}_{\text{a}}[{\text{h}}_{\text{t}}^{\text{dec}},{\text{h}}_{\text{j}}^{\text{enc}}]\right)\right)$ or multiplicative $\left({\text{h}}_{\text{t}}^{{\text{dec}}^{\text{T}}}{\text{W}}_{\text{a}}{\text{h}}_{\text{j}}^{\text{enc}}\right)$, enables the model to focus on relevant input regions dynamically.

By integrating LSTM’s memory retention with attention’s adaptive focus, the hybrid architecture achieves superior performance in tasks requiring fine-grained temporal reasoning, such as outlier detection [27] or financial forecasting [28–30]. The attention weights $\alpha_{\text{tj}}$ also provide interpretable insights into feature importance, bridging the gap between model complexity and transparency. This synergy positions Attention-LSTM as a versatile solution for modern sequence modeling challenges.

In this paper, the Attention-LSTM implementation is configured and tuned with the following hyperparameters: the number of LSTM layers in the encoder: [1, 2], the number of encoder hidden units: [50, 100, 150], the type of attention mechanism: [‘additive’, ‘multiplicative’], the dropout rate: [0.2, 0.3, 0.4], the optimizer: [‘Adam’], the learning rate: [0.0005, 0.001], and the batch size: [16, 32]. We employ early stopping with a patience of 20 epochs. The final model configuration that achieves the best results uses a single LSTM layer with 100 hidden units as the encoder, an additive attention mechanism (Bahdanau), and a dropout rate of 0.3 applied to the LSTM outputs. Training is conducted using the Adam optimizer with a learning rate of 0.0005 and a batch size of 16. The typical training epoch range is 100–150.

#### Support vector regression (SVR).

SVR, an extension of Support Vector Machines (SVMs) proposed by Vapnik et al. [31] in the 1990s, is a supervised learning algorithm designed for regression tasks. Its primary objective is to predict continuous outcomes by identifying a hyperplane that maximizes the margin around predicted values while tolerating small deviations controlled by a parameter ∈. Unlike traditional regression methods that minimize mean squared error, SVR focuses on minimizing the ∈-insensitive loss function, which disregards errors within a predefined tolerance band. This approach enhances robustness to outliers and noisy data, making SVR particularly effective in high-dimensional or non-linear problems, such as financial forecasting and industrial process modeling [32,33].

The core principle of SVR revolves around mapping input data ${\text{x}}_{\text{i}}$ into a higher-dimensional feature space via a kernel function $\text{K}({\text{x}}_{\text{i}},{\text{x}}_{\text{j}})$ (e.g., radial basis function: $\text{K}({\text{x}}_{\text{i}},{\text{x}}_{\text{j}})={\text{e}}^{{-\gamma \left\|{\text{x}}_{\text{i}}-{\text{x}}_{\text{j}}\right\|}^{\text{2}}}$, where a linear regression model is constructed. The optimization objective is formulated as:


$$\begin{array}{c} \text{min}\frac{\text{1}}{\text{2}}{\left\|\text{w}\right\|}^{\text{2}}+\text{C}\sum {\mathrm{\backslash nolimits}}_{\text{i}=\text{1}}^{\text{n}}\left(\xi_{\text{i}}+\xi_{\text{i}}^{*}\right) \end{array}$$ (15)

$$\text{s}.\text{t}.{\text{y}}_{\text{i}}-({\text{w}}^{\text{T}}\emptyset ({\text{x}}_{\text{i}})+\text{b})\leq \epsilon +\xi_{\text{i}}$$ (16)

$$({\text{w}}^{\text{T}}\emptyset ({\text{x}}_{\text{i}})+\text{b})-{\text{y}}_{\text{i}}\leq \epsilon +\xi_{\text{i}}^{*}$$ (17)

$$\xi_{\text{i}},\xi_{\text{i}}^{*}\geq \text{0},\forall \text{i}$$ (18)

where w is the weight vector, C controls the trade-off between margin width and error tolerance, and $\xi_{\text{i}}$, $\xi_{\text{i}}^{*}$ are slack variables. Predictions are made using $\text{f}(\text{x})=\sum_{\text{i}=\text{1}}^{\text{n}}{(\text{a}}_{\text{i}}-{\text{a}}_{\text{i}}^{*}textrmK({\text{x}}_{\text{i}},\text{x})+\text{b}$, where ${\text{a}}_{\text{i}}$, ${\text{a}}_{\text{i}}^{*}$ are Lagrange multipliers. This dual formulation allows SVR to handle non-linearity efficiently without explicitly computing high-dimensional transformations.

In this paper, the SVR model is implemented using the Radial Basis Function (RBF) kernel. Hyperparameters are tuned via grid search on the validation set. The search ranges are: regularization parameter C: [0.1, 1, 10, 100], kernel coefficient γ: [‘scale’, 0.01, 0.1, 1], and tolerance ∊: [0.01, 0.1, 0.5]. The final chosen values that yield the best performance are C = 10, γ = 0.1, and ∊=0.1.

#### Random forest (RF).

Decision trees can be used for various machine learning applications. However, trees grown deep to learn highly irregular patterns tend to overfit the training set. Moreover, a slight noise in the data may cause the tree to grow in a completely different manner.

Random Forest is an upgrade based on the decision trees which is constructed using a combination of decision tree classifiers [34]. Random Forest overcomes this problem by training multiple decision trees on different subspaces of the feature space at the cost of slightly increased bias. This means none of the trees in the forest sees the entire training data. The data is recursively split into partitions. At a particular node, the split is done by asking a question on an attribute. The choice for the splitting criterion is based on some impurity measures such as Shannon Entropy or Gini impurity.

Gini impurity is used as the function to measure the quality of the split in each node. Gini impurity at node N is given by


$$\begin{array}{c} \text{g}(\text{N})=\sum {\mathrm{\backslash nolimits}}_{\text{i}\neq \text{j}}\text{P}\left(\omega_{\text{i}}\right)\text{P}\left(\omega_{\text{j}}\right) \end{array}$$ (19)

where $\text{P}\left(\omega_{\text{i}}\right)$ is the proportion of the population with class label i. Another function that can be used to judge the split quality is Shannon Entropy. It measures the disorder in the information content. In decision trees, Shannon entropy is used to measure the unpredictability in the information contained in a particular node of a tree (In this context, it measures how mixed the population in a node is). The entropy in a node N can be calculated as follows


$$\begin{array}{c} \text{H}(\text{N})=-\sum {\mathrm{\backslash nolimits}}_{\text{i}=\text{1}}^{\text{i}=\text{d}}\text{P}\left(\omega_{\text{i}}\right){\text{log}}_{\text{2}}(\left(\text{P}\left(\omega_{\text{i}}\right)\right) \end{array}$$ (20)

where d is number of classes considered and $\text{P}\left(\omega_{\text{i}}\right)$ is the proportion of the population labeled as i. Entropy is the highest when all the classes are contained in equal proportion in the node. It is the lowest when only one class is present in a node (when the node is pure).

The obvious heuristic approach to choosing the best splitting decision at a node is the one that reduces the impurity as much as possible. In order words, the best split is characterized by the highest gain in information or the highest reduction in impurity. The information gain due to a split can be calculated as follows


$$\Delta \text{I}(\text{N})=\text{I}(\text{N})-{\text{P}}_{\text{L}}\times \text{I}\left({\text{N}}_{\text{L}}\right)-{\text{P}}_{\text{R}}\times \text{I}\left({\text{N}}_{\text{L}}\right)$$ (21)

where I(N) is the impurity measure (Gini or Shannon Entropy) of node N, ${\text{P}}_{\text{L}}$ is the proportion of the population in node N that goes to the left child of N after the split and similarly, ${\text{P}}_{\text{R}}$ is the proportion of the population in node N that goes to the right child after the split. ${\text{N}}_{\text{L}}$ and ${\text{N}}_{\text{R}}$ are the left and right child of N respectively.

Since the whole feature factor subset is no longer considered at a time, but one feature factor subset, the training speed of Random Forest is faster. Besides, each decision tree in Random Forest selects training samples and features randomly, which makes the random forest not easy to fall into overfitting, and also has stronger adaptability to data and can handle discrete and continuous data.

In this paper, the RF model is configured and tuned with the following hyperparameters: the number of trees (n_estimators): [100, 200, 300], the maximum depth of trees (max_depth): [5, 10, 15, None], the minimum samples required to split a node (min_samples_split): [2, 5, 10], and the minimum samples required at a leaf node (min_samples_leaf): [1, 2, 4]. The final model uses 200 trees, a maximum depth of 10, a minimum of 5 samples to split a node, and 2 samples per leaf. The Gini impurity is used as the splitting criterion.

#### Extreme gradient boosting (XGBoost).

XGBoost, proposed by Tianqi Chen and Carlos Guestrin in 2016 [35], is an advanced ensemble learning algorithm designed to optimize predictive accuracy and computational efficiency. Built upon the gradient boosting framework, it addresses limitations in traditional gradient boosting machines (GBMs) by introducing regularization, parallel processing, and hardware optimization. Specifically, the method aims to iteratively combine weak learners (typically decision trees) into a strong ensemble model, minimizing prediction errors while controlling overfitting.

At its core, XGBoost optimizes a regularized objective function L comprising a loss function L (e.g., mean squared error or log loss) and a regularization term φ:


$$\begin{array}{c} L(\emptyset )=\sum {\mathrm{\backslash nolimits}}_{\text{i}=\text{1}}^{\text{n}}\text{L}({\text{y}}_{\text{i}},{\hat{\text{y}}}_{\text{i}})+\sum {\mathrm{\backslash nolimits}}_{\text{k}=\text{1}}^{\text{K}}\varphi ({\text{f}}_{\text{k}}) \end{array}$$ (22)

where ${\hat{\text{y}}}_{\text{i}}=\sum_{\text{k}=\text{1}}^{\text{K}}{\text{f}}_{\text{k}}({\text{x}}_{\text{i}})$ is the ensemble prediction, ${\text{f}}_{\text{k}}$ represents the k-th tree, and $\varphi ({\text{f}}_{\text{k}})=\gamma \text{T}+\frac{\text{1}}{\text{2}}\lambda {\left\|\text{w}\right\|}^{\text{2}}$. Here, T is the number of leaves, w denotes leaf weights, and γ, λ control regularization strength.

During training, trees are added sequentially to correct residuals from previous iterations. The gradient and Hessian of the loss guide tree construction, with the optimal weight ${\text{w}}_{\text{j}}^{*}$ for leaf j computed as:


$${\text{w}}_{\text{j}}^{*}=-\frac{\sum_{i\varepsilon {\text{I}}_{\text{j}}}{\text{g}}_{\text{i}}}{\sum_{i\varepsilon {\text{I}}_{\text{j}}}{\text{h}}_{\text{i}}+\lambda }$$ (23)

where ${\text{g}}_{\text{i}}=\vartheta_{{\text{ij}}^{\left(\text{t}-\text{1}\right)}}\text{L}({\text{y}}_{\text{i}},{\hat{\text{y}}}^{\left(\text{t}-\text{1}\right)})$ and ${\text{h}}_{\text{i}}=\vartheta_{{\text{ij}}^{\left(\text{t}-\text{1}\right)}}^{\text{2}}\text{L}({\text{y}}_{\text{i}},{\hat{\text{y}}}^{\left(\text{t}-\text{1}\right)})$ are first- and second-order gradients.

By balancing model accuracy, computational speed, and interpretability, XGBoost outperforms conventional machine learning methods while avoiding the complexity of deep neural networks. From credit risk modeling to financial prediction, XGBoost’s versatility and efficiency continue to drive its adoption across academia and industry [36–38].

In this paper, the XGBoost model’s hyperparameters are optimized through grid search. Key parameters and their search ranges include: n_estimators: [100, 200, 300], max_depth: [3, 6, 9], learning_rate: [0.01, 0.05, 0.1], subsample: [0.8, 0.9, 1.0], and colsample_bytree: [0.8, 0.9, 1.0], gamma: [0, 0.1, 0.2], and lambda: [1, 1.5, 2]. The final model configuration was: n_estimators = 200, max_depth = 6, learning_rate = 0.05, subsample = 0.9, colsample_bytree = 0.9, gamma = 0.1, and lambda = 1.5.

### Evaluation methods

To assess the predictive ability of the forecasting models, following Lu and Xu [30], and Jiang et al. [39], four main evaluation metrics, out of sample R2, root mean square error (RMSE), mean absolute error (MAE), and mean absolute percentage error (MAPE), are selected. The larger the value of R2, the smaller the values of RMSE, MAE, and MAPE, the better the model performance. The equations of these evaluation metrics are shown below:


$${\text{R}}_{\text{OOS}}^{\text{2}}=\text{1}-\frac{\sum_{\text{t}=\text{m}+\text{1}}^{\text{N}}{\left({\text{y}}_{\text{t}}-{\hat{\text{y}}}_{\text{M}.\text{t}}\right)}^{\text{2}}}{\sum_{\text{t}=\text{m}+\text{1}}^{\text{N}}{\left({\text{y}}_{\text{t}}-{\hat{\text{y}}}_{\text{B}.\text{t}}\right)}^{\text{2}}}$$ (24)

$$\begin{array}{c} \text{RMSE}=\sqrt{\frac{\text{1}}{\text{N}}\sum {\mathrm{\backslash nolimits}}_{\text{t}=\text{1}}^{\text{N}}{\left({\text{y}}_{\text{t}}-{\hat{\text{y}}}_{\text{M}.\text{t}}\right)}^{\text{2}}} \end{array}$$ (25)

$$\begin{array}{c} \text{MAE}=\frac{\text{1}}{\text{N}}\sum {\mathrm{\backslash nolimits}}_{\text{t}=\text{1}}^{\text{N}}\left|{\text{y}}_{\text{t}}-{\hat{\text{y}}}_{\text{M}.\text{t}}\right| \end{array}$$ (26)

$$\begin{array}{c} \text{MAPE}=\frac{\text{1}}{\text{N}}\sum {\mathrm{\backslash nolimits}}_{\text{t}=\text{1}}^{\text{N}}\left|\frac{{\text{y}}_{\text{t}}-{\hat{\text{y}}}_{\text{M}.\text{t}}}{{\text{y}}_{\text{t}}}\right|\times \text{100}\% \end{array}$$ (27)

where ${\text{y}}_{\text{t}}$, ${\hat{\text{y}}}_{\text{M}.\text{t}}$, ${\hat{\text{y}}}_{\text{B}.\text{t}}$ represent the true value, the predicted value of the prediction model, and benchmark prediction of the historical average model at time t, respectively, m is the number of in-sample observations, and N is the number of total samples.

Furthermore, we employ the Diebold–Mariano (DM) test introduced by Diebold and Mariano [40] to evaluate the forecasting performance of the machine learning methods. The null hypothesis of the DM test is that the predictive accuracy of the tested model is equal to the alternative model. Consistent with the previous evaluation step, RMSE, MAE, MAPE are used as the DM loss function, and the historical average model is used as the alternative model, respectively. Given the multiple comparisons inherent in testing across horizons and models, we follow the forecasting literature’s common practice of reporting unadjusted p-values, as our conclusions rely on consistent performance patterns rather than isolated test results, and adjustments would unduly reduce statistical power.

## Empirical results

### LASSO feature selection

Table 2 presents the correlation coefficients among some predictors. The correlation between EUI (EUI_equally, EUI_GDP) and other predictors is small, suggesting that the energy-related uncertainty contains additional information. Moreover, the correlation coefficients of some macroeconomic predictors are shown to be relatively high. Therefore, we need to use the LASSO regression model to select the predictors with strong predictive power, especially the predictors with high correlations.

**Table 2:** Correlation coefficients among predictors.

	LIBOR	LTY	DJIA	SVAR	GSCI-NE	UR	CFNAI	INFL	MS	DOLLAR	MSCI	GCOP	GCOI	EUI_equally	EUI_GDP
LIBOR	1.000														
LTY	0.841	1.000													
DJIA	0.429	0.687	1.000												
GSCI-NE	0.607	0.696	0.586	0.019	1.000										
UR	0.531	0.327	0.222	0.081	0.320	1.000									
CFNAI	0.011	0.067	0.062	0.327	0.009	0.160	1.000								
INFL	0.726	0.899	0.813	0.020	0.809	0.165	0.046	1.000							
MS	0.634	0.842	0.950	0.000	0.691	0.029	0.016	0.912	1.000						
DOLLAR	0.035	0.116	0.478	0.117	0.284	0.461	0.038	0.161	0.378	1.000					
MSCI	0.337	0.620	0.983	0.045	0.572	0.275	0.074	0.776	0.913	0.466	1.000				
GCOP	0.660	0.861	0.800	0.013	0.731	0.021	0.075	0.961	0.872	0.179	0.759	1.000			
GCOI	0.328	0.462	0.643	0.033	0.362	0.013	0.021	0.417	0.650	0.251	0.602	0.427	1.000		
EUI_equally	0.334	0.116	0.037	0.046	0.213	0.355	0.091	0.169	0.030	0.220	0.044	0.058	0.200	1.000	
EUI_GDP	0.328	0.506	0.334	0.095	0.221	0.042	0.170	0.493	0.394	0.284	0.306	0.519	0.049	0.326	1.000

By adopting the LassoCV method, we are able to choose the optimal value of penalty coefficient λ, which is set to be [0.01, 0.05, 0.1, 0.2, 0.3, 0.4, 0.5, 1]. Table 3 shows the predictors we selected through the LASSO regression method, except EUI_equally and EUI_GDP.

**Table 3:** Selected factors via LASSO.

Factor category	Selected factors
Bond market	TBL
	DFY
	LTY
Stock market	SVAR
Futures market	GSCI-NE
	CRB
Economic state	CU
	CFNAI
	INFL
	MS
	IPI
	DOLLAR
	ISM
Related crude oil market	USCOP
	USCOS
	GCOI
	USCOI

### One-month-ahead forecasting results

In this subsection, we run 6 × 3 models, namely, six prediction methods (LR, LSTM, Attention-LSTM, SVR, RF, XGBoost), with three energy uncertainty indices (EUI_equally, EUI_GDP, EUI_without), to predict crude oil prices after one month. We use the adam optimizer and RMSE loss function for all machine learning methods. Other parameters that need to be tuned, such as the kernel and the value of epsilon for the SVR model, are determined through the trial and error method. All experiments are implemented in Python 3.7.

Table 4 shows the one-month-ahead forecasting results, Tables 5 and 6 present the improvement percentages of the EUI index and the best machine learning model, respectively. It can be concluded that the RF model with EUI is superior to other models in the short term. Specifically, it can be summarized as follows:

**Table 4:** One-month-ahead forecasting performance for all models.

Models	Evaluation metrics	EUI_without	EUI_equally	EUI_GDP
LR	${\text{R}}_{\text{OOS}}^{\text{2}}$	0.235	0.299	0.270
	RMSE	26.599***	26.270***	26.553***
	MAE	20.177***	19.757***	20.138***
	MAPE	0.305***	0.298***	0.304***
LSTM	${\text{R}}_{\text{OOS}}^{\text{2}}$	0.585	0.619	0.622
	RMSE	17.003***	16.287***	16.236***
	MAE	14.221***	13.678***	13.573***
	MAPE	0.244***	0.231***	0.227***
Attention-LSTM	${\text{R}}_{\text{OOS}}^{\text{2}}$	0.596	0.623	0.639
	RMSE	16.507***	16.040***	15.699***
	MAE	14.045***	13.221***	12.142***
	MAPE	0.233***	0.226***	0.218***
SVR	${\text{R}}_{\text{OOS}}^{\text{2}}$	0.207	0.227	0.218
	RMSE	30.397***	28.888***	29.532***
	MAE	25.858***	24.986***	25.206***
	MAPE	0.491***	0.463***	0.477***
RF	${\text{R}}_{\text{OOS}}^{\text{2}}$	**0.658**	**0.665**	**0.664**
	RMSE	15.388***	15.219***	15.252***
	MAE	11.818***	11.641***	11.427***
	MAPE	0.214***	0.211***	0.212***
XGBoost	${\text{R}}_{\text{OOS}}^{\text{2}}$	0.631	0.653	0.637
	RMSE	15.967***	15.489***	15.833***
	MAE	12.376***	12.005***	12.215***
	MAPE	0.226***	0.216***	0.221***

Note: *, **, and *** represent statistical significance at the 10%, 5%, and 1% levels, respectively.

**Table 5:** Improvement percentages of the EUI index in one-month-ahead forecasting.

Models	Evaluation metrics	EUI_equally	EUI_GDP
LR	${\text{R}}_{\text{OOS}}^{\text{2}}$	27.234%	14.894%
	RMSE	1.252%	0.173%
	MAE	2.126%	0.194%
	MAPE	2.349%	0.329%
LSTM	${\text{R}}_{\text{OOS}}^{\text{2}}$	5.812%	6.325%
	RMSE	4.396%	4.724%
	MAE	3.970%	4.774%
	MAPE	5.628%	7.489%
Attention-LSTM	${\text{R}}_{\text{OOS}}^{\text{2}}$	4.530%	7.215%
	RMSE	2.911%	5.147%
	MAE	6.233%	15.673%
	MAPE	3.097%	6.881%
SVR	${\text{R}}_{\text{OOS}}^{\text{2}}$	9.662%	5.314%
	RMSE	5.224%	2.929%
	MAE	3.490%	2.587%
	MAPE	6.048%	2.935%
RF	${\text{R}}_{\text{OOS}}^{\text{2}}$	1.064%	0.912%
	RMSE	1.110%	0.892%
	MAE	1.520%	3.422%
	MAPE	1.422%	0.943%
XGBoost	${\text{R}}_{\text{OOS}}^{\text{2}}$	3.487%	0.951%
	RMSE	3.086%	0.846%
	MAE	3.090%	1.318%
	MAPE	4.630%	2.262%

**Table 6:** Improvement percentages of the RF model.

Comparative models	Evaluation metrics	EUI_without	EUI_equally	EUI_GDP
LR	${\text{R}}_{\text{OOS}}^{\text{2}}$	180.000%	122.408%	145.926%
	RMSE	72.855%	72.613%	74.095%
	MAE	70.731%	69.719%	76.232%
	MAPE	42.523%	41.232%	43.396%
LSTM	${\text{R}}_{\text{OOS}}^{\text{2}}$	12.479%	7.431%	6.752%
	RMSE	10.495%	7.018%	6.452%
	MAE	20.333%	17.498%	18.780%
	MAPE	14.019%	9.479%	7.075%
Attention-LSTM	${\text{R}}_{\text{OOS}}^{\text{2}}$	10.403%	6.742%	3.912%
	RMSE	7.272%	5.395%	2.931%
	MAE	18.844%	13.573%	6.257%
	MAPE	8.879%	7.109%	2.830%
SVR	${\text{R}}_{\text{OOS}}^{\text{2}}$	217.874%	192.952%	204.587%
	RMSE	97.537%	89.815%	93.627%
	MAE	118.802%	114.638%	120.583%
	MAPE	129.439%	119.431%	125.000%
XGBoost	${\text{R}}_{\text{OOS}}^{\text{2}}$	4.279%	1.838%	4.239%
	RMSE	3.763%	1.774%	3.809%
	MAE	4.722%	3.127%	6.896%
	MAPE	5.607%	2.370%	4.245%

(1) The inclusion of energy uncertainty index (EUI) can improve the predictive accuracy of all models, as shown in Tables 4 and 5. For instance, using the RF model, the EUI_equally can improve the performance of ${\text{R}}_{\text{OOS}}^{\text{2}}$, RMSE, MAE, MAPE by 1.064%, 1.110%, 1.520%, 1.422% compared to the RF model using no EUI, respectively. The EUI_GDP can improve the performance of ${\text{R}}_{\text{OOS}}^{\text{2}}$, RMSE, MAE, MAPE by 0.912%, 0.892%, 3.422%, 0.943% compared to the RF model using no EUI, respectively.
(2) Based on the DM test reported in Table 4, all prediction models are helpful in predicting crude oil price, and the RF models outperform other models in the short term. Here, the results of the DM test are indicated with an asterisk. According to Table 4, all prediction models perform better than the baseline model, which is a historical average model in this study. Moreover, according to Table 6, no matter which EUI is used in the prediction models, the RF models always perform better than others For example, using the EUI_equally in the models, the RF model can improve the performance of ${\text{R}}_{\text{OOS}}^{\text{2}}$ by 122.408%, 7.431%, 6.742%, 192.952%, 1.838% compared to LR, LSTM, Attention-LSTM, SVR, RF, XGBoost models, respectively. Using the EUI_GDP in the models, the RF model can improve the performance of ${\text{R}}_{\text{OOS}}^{\text{2}}$ by 145.926%, 6.752%, 3.912%, 204.587%, 4.239% compared to LR, LSTM, Attention-LSTM, SVR, XGBoost models, respectively. Similar conclusions can be drawn using other evaluation metrics.

Combining the results summarized above, it is concluded that the RF model with EUI is superior to other types of models in the short term. We attribute this advantage to two interrelated factors: the intrinsic capabilities of the Random Forest algorithm and the distinct role of EUI in high-frequency price dynamics. RF excels in capturing the complex, nonlinear interactions among transient market features—such as inventory shocks, speculative flows, and currency fluctuations—through its ensemble structure and feature selection mechanism, which effectively filters high-frequency noise while identifying the most relevant short-term drivers.

In this context, EUI functions as a critical barometer of immediate market sentiment and energy-specific risk. It captures real-time uncertainty stemming from geopolitical events, supply disruptions, or sudden policy shifts in the energy sector, which often trigger rapid repricing in oil futures. By incorporating EUI, the RF model gains access to a high-frequency proxy for the “fear factor” that drives short-term volatility—a dimension often missed by conventional macroeconomic variables. Thus, the predictive superiority of RF with EUI not only reflects the model’s algorithmic strength but also underscores the essential role of energy uncertainty as a near-term risk amplifier in crude oil markets.

### Three-month-ahead forecasting results

In this subsection, we conduct a three-month-ahead prediction to further investigate the impact of EUI on the crude oil market in the long term. The empirical results presented in Tables 7–9 show that the Attention-LSTM model with EUI is superior to other competing models over this horizon. This can be explained by the model’s architectural strengths in capturing temporal dependencies and the evolving nature of EUI’s influence beyond short-term noise.

**Table 7:** Three-month-ahead forecasting performance for all models.

Models	Evaluation metrics	EUI_without	EUI_equally	EUI_GDP
LR	${\text{R}}_{\text{OOS}}^{\text{2}}$	0.174	0.217	0.240
	RMSE	42.655***	41.053***	40.909***
	MAE	35.559***	33.838***	33.689***
	MAPE	0.639***	0.608***	0.605***
LSTM	${\text{R}}_{\text{OOS}}^{\text{2}}$	0.583	0.646	0.622
	RMSE	16.908***	15.591***	16.097***
	MAE	13.217***	12.901***	13.089***
	MAPE	0.244***	0.219***	0.233***
Attention-LSTM	${\text{R}}_{\text{OOS}}^{\text{2}}$	**0.629**	**0.651**	**0.683**
	RMSE	15.681***	15.191***	13.206***
	MAE	12.987***	12.089***	11.082***
	MAPE	0.226***	0.216***	0.201***
SVR	${\text{R}}_{\text{OOS}}^{\text{2}}$	0.309	0.328	0.354
	RMSE	30.964***	29.211***	27.706***
	MAE	26.316***	24.877***	23.372***
	MAPE	0.492***	0.470***	0.441***
RF	${\text{R}}_{\text{OOS}}^{\text{2}}$	0.565	0.570	0.582
	RMSE	17.198***	17.111***	16.863***
	MAE	14.249***	12.903***	13.314***
	MAPE	0.267***	0.221***	0.247***
XGBoost	${\text{R}}_{\text{OOS}}^{\text{2}}$	0.455	0.473	0.520
	RMSE	19.250***	18.942***	18.073***
	MAE	14.888***	14.864***	13.878***
	MAPE	0.282***	0.280***	0.256***

Note: *, **, and *** represent statistical significance at the 10%, 5%, and 1% levels, respectively.

**Table 8:** Improvement percentages of the EUI index in three-month-ahead forecasting.

Models	Evaluation metrics	EUI_equally	EUI_GDP
LR	${\text{R}}_{\text{OOS}}^{\text{2}}$	24.713%	37.931%
	RMSE	3.902%	4.268%
	MAE	5.086%	5.551%
	MAPE	5.099%	5.620%
LSTM	${\text{R}}_{\text{OOS}}^{\text{2}}$	10.806%	6.690%
	RMSE	8.447%	5.038%
	MAE	2.449%	0.978%
	MAPE	11.416%	4.721%
Attention-LSTM	${\text{R}}_{\text{OOS}}^{\text{2}}$	3.498%	8.585%
	RMSE	3.226%	18.741%
	MAE	7.428%	17.190%
	MAPE	4.630%	12.438%
SVR	${\text{R}}_{\text{OOS}}^{\text{2}}$	6.149%	14.563%
	RMSE	6.001%	11.759%
	MAE	5.784%	12.596%
	MAPE	4.681%	11.565%
RF	${\text{R}}_{\text{OOS}}^{\text{2}}$	0.885%	3.009%
	RMSE	0.508%	1.987%
	MAE	10.432%	7.023%
	MAPE	20.814%	8.097%
XGBoost	${\text{R}}_{\text{OOS}}^{\text{2}}$	3.956%	14.286%
	RMSE	1.626%	6.512%
	MAE	0.161%	7.278%
	MAPE	0.714%	10.156%

**Table 9:** Improvement percentages of the LSTM model in three-month-ahead forecasting.

Comparative models	Evaluation metrics	EUI_without	EUI_equally	EUI_GDP
LR	${\text{R}}_{\text{OOS}}^{\text{2}}$	261.494%	200.000%	184.583%
	RMSE	172.017%	170.246%	209.776%
	MAE	173.805%	179.907%	203.997%
	MAPE	182.743%	181.481%	200.995%
LSTM	${\text{R}}_{\text{OOS}}^{\text{2}}$	7.890%	0.774%	9.807%
	RMSE	7.825%	2.633%	21.892%
	MAE	1.771%	6.717%	18.110%
	MAPE	7.965%	1.389%	15.920%
SVR	${\text{R}}_{\text{OOS}}^{\text{2}}$	103.560%	73.780%	64.407%
	RMSE	97.462%	92.291%	109.799%
	MAE	102.633%	105.782%	110.901%
	MAPE	117.699%	117.593%	119.403%
RF	${\text{R}}_{\text{OOS}}^{\text{2}}$	11.327%	14.211%	17.354%
	RMSE	9.674%	12.639%	27.692%
	MAE	9.717%	6.733%	20.141%
	MAPE	18.142%	2.315%	22.886%
XGBoost	${\text{R}}_{\text{OOS}}^{\text{2}}$	38.242%	37.632%	31.346%
	RMSE	22.760%	24.692%	36.854%
	MAE	14.638%	22.955%	25.230%
	MAPE	24.779%	29.630%	27.363%

Specifically, the Attention-LSTM combines the long-range memory of LSTM with a dynamic attention mechanism, enabling it to identify and weigh historically significant periods—such as sustained uncertainty regimes or structural breaks—that shape medium- to long-term price trends. In such a framework, EUI transitions from being a high-frequency sentiment indicator to a forward-looking gauge of fundamental risks affecting investment, production, and consumption decisions. Persistent rise in EUI may signal delays in oilfield development, energy transition, shifts in the global political landscape, or policy uncertainty that gradually alters supply-demand balances. The Attention-LSTM model, by assigning learned importance to relevant past states of EUI and its interactions with slow-moving fundamentals, effectively captures how energy uncertainty propagates into long-term price dynamics. Therefore, the outperformance of Attention-LSTM with EUI not only demonstrates its technical merit but also validates EUI’s role as a persistent driver in the formation of medium-term crude oil price trends.

### Six-month-ahead forecasting results

To validate the results in the long term, we conduct the six-month-ahead prediction in this subsection. The empirical results are shown in Tables 10–12. As shown in Tables 10–12, the Attention-LSTM model with EUI still outperforms other models in the long run. These results are consistent with the results in the above section.

**Table 10:** Six-month-ahead forecasting performance for all models.

Models	Evaluation metrics	EUI_without	EUI_equally	EUI_GDP
LR	${\text{R}}_{\text{OOS}}^{\text{2}}$	0.064	0.079	0.099
	RMSE	214.843***	211.515***	209.443***
	MAE	183.655***	180.534***	178.744***
	MAPE	3.047***	2.993***	2.964***
LSTM	${\text{R}}_{\text{OOS}}^{\text{2}}$	0.657	0.663	0.671
	RMSE	15.162***	15.025***	14.854***
	MAE	12.778***	12.684***	12.434***
	MAPE	0.228***	0.224***	0.221***
Attention-LSTM	${\text{R}}_{\text{OOS}}^{\text{2}}$	**0.675**	**0.695**	**0.686**
	RMSE	14.796***	13.167***	13.921***
	MAE	12.153***	10.941***	11.837***
	MAPE	0.219***	0.213***	0.215***
SVR	${\text{R}}_{\text{OOS}}^{\text{2}}$	0.284	0.309	0.299
	RMSE	38.966***	37.230***	38.154***
	MAE	33.270***	31.534***	32.562***
	MAPE	0.638***	0.606***	0.624***
RF	${\text{R}}_{\text{OOS}}^{\text{2}}$	0.312	0.374	0.350
	RMSE	35.654***	28.213***	31.584***
	MAE	29.741***	23.903***	26.799***
	MAPE	0.588***	0.465***	0.522***
XGBoost	${\text{R}}_{\text{OOS}}^{\text{2}}$	0.362	0.410	0.390
	RMSE	29.284***	23.616***	27.796***
	MAE	24.855***	18.907***	23.138***
	MAPE	0.461***	0.345***	0.432***

Note: *, **, and *** represent statistical significance at the 10%, 5%, and 1% levels, respectively.

**Table 11:** Improvement percentages of the EUI index in six-month-ahead forecasting.

Models	Evaluation metrics	EUI_equally	EUI_GDP
LR	${\text{R}}_{\text{OOS}}^{\text{2}}$	23.438%	54.688%
	RMSE	1.573%	2.578%
	MAE	1.729%	2.748%
	MAPE	1.804%	2.800%
LSTM	${\text{R}}_{\text{OOS}}^{\text{2}}$	0.913%	2.131%
	RMSE	0.912%	2.074%
	MAE	0.741%	2.767%
	MAPE	1.786%	3.167%
Attention-LSTM	${\text{R}}_{\text{OOS}}^{\text{2}}$	2.963%	1.630%
	RMSE	12.372%	6.285%
	MAE	11.078%	2.670%
	MAPE	2.817%	1.860%
SVR	${\text{R}}_{\text{OOS}}^{\text{2}}$	8.803%	5.282%
	RMSE	4.663%	2.128%
	MAE	5.505%	2.174%
	MAPE	5.281%	2.244%
RF	${\text{R}}_{\text{OOS}}^{\text{2}}$	19.872%	12.179%
	RMSE	26.374%	12.886%
	MAE	24.424%	10.978%
	MAPE	26.452%	12.644%
XGBoost	${\text{R}}_{\text{OOS}}^{\text{2}}$	13.260%	7.735%
	RMSE	24.001%	5.353%
	MAE	31.459%	7.421%
	MAPE	33.623%	6.713%

**Table 12:** Improvement percentages of the LSTM model in six-month-ahead forecasting.

Comparative models	Evaluation metrics	EUI_without	EUI_equally	EUI_GDP
LR	${\text{R}}_{\text{OOS}}^{\text{2}}$	954.688%	779.747%	592.929%
	RMSE	1352.034%	1506.402%	1404.511%
	MAE	1411.191%	1550.069%	1410.045%
	MAPE	1291.324%	1305.164%	1278.605%
LSTM	${\text{R}}_{\text{OOS}}^{\text{2}}$	2.740%	4.827%	2.235%
	RMSE	2.474%	14.111%	6.702%
	MAE	5.143%	15.931%	5.044%
	MAPE	4.110%	5.164%	2.791%
SVR	${\text{R}}_{\text{OOS}}^{\text{2}}$	137.676%	21.036%	17.057%
	RMSE	163.355%	182.752%	174.075%
	MAE	173.760%	188.219%	175.087%
	MAPE	191.324%	184.507%	190.233%
RF	${\text{R}}_{\text{OOS}}^{\text{2}}$	116.346%	85.829%	96.000%
	RMSE	140.971%	114.271%	126.880%
	MAE	144.721%	118.472%	126.400%
	MAPE	168.493%	118.310%	142.791%
XGBoost	${\text{R}}_{\text{OOS}}^{\text{2}}$	86.464%	69.512%	75.897%
	RMSE	97.918%	79.357%	99.670%
	MAE	104.517%	72.809%	95.472%
	MAPE	110.502%	61.972%	100.930%

### Twelve-month-ahead forecasting results

To further validate the results in the long term, we conduct the twelve-month-ahead prediction in this subsection. The empirical results are shown in Tables 13–15. As shown in Tables 13–15, the Attention-LSTM model with EUI remains superior to the other models in the long term. These results are consistent with the results in the above section.

**Table 13:** Twelve-month-ahead forecasting performance for all models.

Models	Evaluation metrics	EUI_without	EUI_equally	EUI_GDP
LR	${\text{R}}_{\text{OOS}}^{\text{2}}$	0.072	0.093	0.085
	RMSE	285.844***	281.252***	284.928***
	MAE	245.888***	241.939***	245.072***
	MAPE	4.082***	4.018***	4.068***
LSTM	${\text{R}}_{\text{OOS}}^{\text{2}}$	0.536	0.548	0.610
	RMSE	17.409***	17.178***	15.965***
	MAE	14.806***	14.001***	13.326***
	MAPE	0.257***	0.246***	0.229***
Attention-LSTM	${\text{R}}_{\text{OOS}}^{\text{2}}$	**0.627**	**0.685**	**0.687**
	RMSE	15.606***	14.336***	14.306***
	MAE	12.688***	12.216***	11.694***
	MAPE	0.221***	0.208***	0.199***
SVR	${\text{R}}_{\text{OOS}}^{\text{2}}$	0.188	0.223	0.203
	RMSE	61.769***	60.092***	60.253***
	MAE	54.370***	52.710***	52.918***
	MAPE	0.991***	0.961***	0.965***
RF	${\text{R}}_{\text{OOS}}^{\text{2}}$	0.295	0.303	0.305
	RMSE	28.747***	28.567***	28.475***
	MAE	25.928***	25.770***	25.688***
	MAPE	0.475***	0.471***	0.469***
XGBoost	${\text{R}}_{\text{OOS}}^{\text{2}}$	0.266	0.274	0.289
	RMSE	42.336***	38.049***	34.985***
	MAE	35.509***	31.809***	29.181***
	MAPE	0.701***	0.630***	0.578***

Note: *, **, and *** represent statistical significance at the 10%, 5%, and 1% levels, respectively.

**Table 14:** Improvement percentages of the EUI index in twelve-month-ahead forecasting.

Models	Evaluation metrics	EUI_equally	EUI_GDP
LR	${\text{R}}_{\text{OOS}}^{\text{2}}$	29.473%	18.288%
	RMSE	1.633%	0.321%
	MAE	1.632%	0.333%
	MAPE	1.586%	0.340%
LSTM	${\text{R}}_{\text{OOS}}^{\text{2}}$	2.281%	13.767%
	RMSE	1.345%	9.048%
	MAE	5.747%	11.105%
	MAPE	4.493%	12.190%
Attention-LSTM	${\text{R}}_{\text{OOS}}^{\text{2}}$	9.276%	9.489%
	RMSE	8.854%	9.086%
	MAE	3.863%	8.505%
	MAPE	5.994%	10.769%
SVR	${\text{R}}_{\text{OOS}}^{\text{2}}$	18.433%	7.727%
	RMSE	2.790%	2.516%
	MAE	3.150%	2.744%
	MAPE	3.052%	2.622%
RF	${\text{R}}_{\text{OOS}}^{\text{2}}$	2.458%	3.316%
	RMSE	0.632%	0.957%
	MAE	0.615%	0.937%
	MAPE	0.880%	1.343%
XGBoost	${\text{R}}_{\text{OOS}}^{\text{2}}$	3.099%	8.595%
	RMSE	11.266%	21.012%
	MAE	11.631%	21.685%
	MAPE	11.319%	21.315%

**Table 15:** Improvement percentages of the LSTM model in twelve-month-ahead forecasting.

Comparative models	Evaluation metrics	EUI_without	EUI_equally	EUI_GDP
LR	${\text{R}}_{\text{OOS}}^{\text{2}}$	769.283%	633.677%	704.625%
	RMSE	1731.661%	1861.797%	1891.678%
	MAE	1837.892%	1880.433%	1995.740%
	MAPE	1750.026%	1830.316%	1942.310%
LSTM	${\text{R}}_{\text{OOS}}^{\text{2}}$	17.003%	25.004%	12.604%
	RMSE	11.556%	19.822%	11.595%
	MAE	16.688%	14.609%	13.958%
	MAPE	16.653%	18.329%	15.175%
SVR	${\text{R}}_{\text{OOS}}^{\text{2}}$	233.525%	35.867%	50.620%
	RMSE	295.810%	319.157%	321.176%
	MAE	328.502%	331.465%	352.531%
	MAPE	349.017%	361.838%	384.666%
RF	${\text{R}}_{\text{OOS}}^{\text{2}}$	112.368%	126.499%	125.057%
	RMSE	84.210%	99.260%	99.043%
	MAE	104.345%	110.943%	119.668%
	MAPE	115.372%	126.292%	135.405%
XGBoost	${\text{R}}_{\text{OOS}}^{\text{2}}$	135.833%	149.963%	137.775%
	RMSE	171.283%	165.402%	144.547%
	MAE	179.849%	160.377%	149.538%
	MAPE	217.654%	202.459%	190.040%

### Forecasting uncertainty and interval analysis

To address the uncertainty surrounding our point prediction and enhance the practical relevance of our findings, we supplement our analysis with an evaluation of prediction intervals. Following the common practice in previous literature [41,42], we employ the residual-based bootstrapping method to construct empirical prediction intervals for our best-performing models: the RF model for one-month-ahead forecasts and the Attention-LSTM model for three-, six-, and twelve-month-ahead forecasts.

The procedure is as follows: First, we fit the model with the training set. Second, after obtaining the final model, we calculate the distribution of residuals on the training set. Third, for each point forecast in the test set, we generate 1000 bootstrap samples by adding randomly sampled residuals to the point forecast. Forth, we form the 95% empirical prediction interval with the 2.5th and 97.5th percentiles of this bootstrap distribution. Fifth, We assess the quality of these intervals by their coverage rate—the percentage of testing set that fall within the constructed interval. The results are summarized in Table 16.

**Table 16:** Prediction Interval Coverage Rates for Best Models.

Model	Horizon (month)	EUI Variant	Nominal Coverage	Empirical Coverage Rate
RF	1-month	EUI_equally	95%	94.627%
		EUI_GDP	95%	94.441%
Attention-LSTM	3-month	EUI_equally	95%	92.429%
		EUI_GDP	95%	92.791%
	6-month	EUI_equally	95%	92.208%
		EUI_GDP	95%	91.908%
	12-month	EUI_equally	95%	92.839%
		EUI_GDP	95%	92.681%

As shown in Table 16, the empirical coverage rates for these models are close to the nominal 95% level. This indicates that the bootstrapped prediction intervals are well-calibrated and reliably capture the forecast uncertainty. The slight under-coverage, particularly for the longer-horizon Attention-LSTM model, is common in financial forecasting and reflects the challenge of encapsulating all sources of uncertainty in highly volatile markets.

### Feature importance analysis

In the previous section, LASSO regression is utilized for feature selection. In this subsection, we use other advanced methods including SHAP (SHapleyAdditive exPlanations) values and attention scores to further investigate the feature importance in each horizon. Specifically, in the 1-month-ahead prediction, we use RF model and SHAP values to sort the selected features. In the 3-month-ahead, 6-month-ahead, 12-month-ahead predictions, we use Attention-LSTM model and attention scores to rank these features. The results are shown in Table 17, in which the top seven features are sorted in descending order.

**Table 17:** Feature importance of crude oil futures prices.

Rank	Horizon (month)			
	1	3	6	12
1	USCOS	CU	IPI	DFY
2	USCOP	INFL	TBL	LTY
3	SVAR	LTY	USCOI	INFL
4	ISM	CRB	GSCI-NE	CRB
5	DOLLAR	MS	DOLLAR	CU
6	GSCI-NE	GCOI	ISM	IPI
7	CFNAI	DFY	MS	GCOI

The feature importance rankings in Table 16 reflect a clear temporal hierarchy, where short-term oil price dynamics (1-month horizon) are dominated by immediate supply-demand imbalances and market sentiment. The top-ranked feature, Growth of US crude oil stock, directly captures inventory fluctuations that signal near-term supply tightness or surplus, while Growth of US crude oil production reflects shale operators’ rapid response to price signals, both driving short-term price volatility. Moreover, high-frequency financial indicators like stock variance and the ISM manufacturing index further amplify these effects by quantifying speculative trading intensity and real-time industrial demand shocks. Concurrently, the US dollar index and S&P GSCI Non-Energy index introduce short-term noise through currency valuation shifts and cross-commodity arbitrage, respectively. These features collectively highlight the dominance of operational and transactional factors over structural and macroeconomic policy drivers in shore-term forecasting.

As the forecasting horizon extends to 3–12 months, structural economic indicators and policy anticipation supersede transient supply shocks. For instance, capacity utilization and inflation rate emerge as critical drivers at the 3-month horizon, which gradually reshape energy investment and consumption patterns. At the 6-month horizon, lagged effects of systemic variables and monetary policy such as industrial production index and treasury bill rates, gain prominence, reflecting the delayed transmission of global trade adjustments and credit conditions. Over 12 months, features such as default yield spread and CRB Rind index dominate, as they encapsulate macroeconomic stability risks and commodity supercycle trends. This transition from operational and transactional features to structural and policy drivers underscores the increasing relevance of macroeconomic stability, policy cycles, and structural demand rebalancing in longer-term predictions.

## Robustness tests

### Different feature selection method

The out-of-sample performance is sensitive to feature selection methods. In this subsection, we consider Random Forest (RF) as an alternative feature selection method in crude oil price prediction. We first fit a RF model on the training set to obtain feature importance scores, then select the top 17 features to form the modified predictor set. The number of features is set to match that chosen by LASSO in the main analysis. The data splitting scheme remains strictly chronological—training (60%), validation (20%), and testing (20%)—to prevent look-ahead bias. The evaluation metrics is RMSE in this subsection and the prediction results are shown in Table 18. The results provide strong empirical evidence that the RF model with EUI outperforms other models in the short term (one-month-ahead), while the Attention-LSTM model with EUI is superior to other models in the long term (three-month-ahead and longer). These results are consistent with previous findings.

**Table 18:** Forecasting performance using RF method.

Models	Horizon (month)	EUI_without	EUI_equally	EUI_GDP
LR	1	39.208***	38.225***	38.144***
	3	115.544***	113.803***	114.599***
	6	205.838***	203.130***	205.015***
	12	171.493***	170.176***	169.964***
LSTM	1	17.945***	17.359***	17.493***
	3	18.389***	18.099***	17.866***
	6	15.992***	15.982***	15.773***
	12	17.651***	16.129***	17.000***
Attention-LSTM	1	17.512***	17.122***	16.885***
	3	18.275***	17.822***	17.283***
	6	15.863***	15.436***	15.211***
	12	17.207***	16.025***	15.814***
SVR	1	30.251***	25.747***	25.721***
	3	51.294***	36.781***	37.729***
	6	36.924***	36.917***	32.853***
	12	43.604***	36.537***	39.837***
RF	1	17.046***	16.208***	16.059***
	3	19.625***	18.568***	17.552***
	6	39.724***	30.723***	33.392***
	12	28.926***	27.448***	28.582***
XGBoost	1	18.127***	17.366***	17.518***
	3	20.513***	20.503***	20.183***
	6	33.938***	30.210***	28.216***
	12	32.167***	30.719***	32.019***

Note: *, **, and *** represent statistical significance at the 10%, 5%, and 1% levels, respectively.

### Different crude oil futures

In the crude oil market, Brent crude oil and WTI crude oil are two essential varieties that attract a lot of attention. They are not only the benchmark for global oil pricing but also the investments to which investors pay close attention. In this subsection, we re-examine the forecasting performance of the RF model with EUI and the Attention-LSTM model with EUI by considering WTI crude oil futures, respectively. The data of WTI crude oil futures price is collected from EIA. The WTI series ranges from February 1996 to October 2022, containing 321 months. The same set of predictors selected by LASSO in the main analysis is used, and the identical chronological split is applied to the WTI data. The evaluation metrics are also RMSE, and the prediction results are shown in Table 19. Obviously, the RF model with EUI can yield smaller RMSE in the short term, while the Attention-LSTM model with EUI has higher predictive accuracy in the long term. These results are consistent with previous results.

**Table 19:** Forecasting performance using WTI crude oil futures.

Models	Horizon (month)	EUI_without	EUI_equally	EUI_GDP
LR	1	75.536***	74.201***	74.145***
	3	43.794***	43.692***	40.765***
	6	167.008***	166.638***	160.228***
	12	165.400***	161.643***	165.273***
LSTM	1	16.861***	16.053***	16.344***
	3	15.343***	14.388***	15.018***
	6	16.704***	16.140***	16.480***
	12	16.414***	14.476***	15.894***
Attention-LSTM	1	16.812***	16.028***	16.192***
	3	15.218***	14.174***	14.526***
	6	16.235***	15.726***	15.961***
	12	15.811***	13.819***	14.739***
SVR	1	20.789***	20.313***	19.658***
	3	29.510***	25.366***	24.690***
	6	30.507***	29.220***	30.033***
	12	33.664***	33.245***	32.334***
RF	1	16.780***	15.176***	15.064***
	3	22.636***	20.002***	20.549***
	6	25.413***	25.005***	25.327***
	12	47.379***	37.035***	28.049***
XGBoost	1	17.883***	17.253***	17.865***
	3	19.382***	19.306***	19.084***
	6	42.571***	39.979***	41.348***
	12	32.446***	31.602***	31.012***

Note: *, **, and *** represent statistical significance at the 10%, 5%, and 1% levels, respectively.

### Different data splits

While our primary analysis employs a fixed 60/20/20 chronological split, we acknowledge that the choice of split ratio may influence model performance. To assess the sensitivity of our findings, we conduct additional experiments using a 70%/15%/15% chronological split. This configuration allows us to examine whether our core conclusions remain stable under different data allocations. Using the same LASSO-selected predictors and RMSE as the evaluation metric, the results summarized in Table 20 demonstrate remarkable consistency. Specifically, RF with EUI maintains superior performance for one-month-ahead forecasts, while Attention-LSTM with EUI continues to achieve the lowest RMSE for three-, six-, and twelve-month-ahead forecasts.

**Table 20:** Forecasting performance under different data split.

Models	Horizon (month)	EUI_without	EUI_equally	EUI_GDP
LR	1	17.763***	17.701***	17.701***
	3	23.604***	23.602***	23.471***
	6	37.473***	37.459***	36.799***
	12	43.665***	43.542***	43.293***
LSTM	1	31.503***	28.847***	28.847***
	3	23.823***	23.622***	21.065***
	6	22.197***	20.904***	20.967***
	12	25.912***	21.846***	23.747***
Attention-LSTM	1	30.517***	28.252***	28.252***
	3	21.816***	17.562***	16.539***
	6	22.144***	20.485***	20.437***
	12	25.223***	19.443***	20.497***
SVR	1	25.102***	19.949***	19.949***
	3	26.069***	18.517***	20.651***
	6	33.277***	32.200***	32.002***
	12	32.006***	30.650***	30.088***
RF	1	15.948***	12.714***	12.714***
	3	24.790***	20.313***	23.527***
	6	26.785***	25.693***	26.721***
	12	26.490***	23.139***	22.480***
XGBoost	1	25.522***	24.553***	25.522***
	3	25.497***	25.096***	24.477***
	6	36.678***	36.422***	34.685***
	12	31.803***	28.832***	30.272***

Note: *, **, and *** represent statistical significance at the 10%, 5%, and 1% levels, respectively.

### Different forecasting target

To address concerns that our findings might be driven by the persistence of crude oil price levels rather than genuine predictive relationships, we conduct a robustness check by forecasting returns instead of price levels We define returns as the logarithmic first difference: ${\text{r}}_{\text{t}}=\text{log}({\text{P}}_{\text{t}})-\text{log}({\text{P}}_{\text{t}-\text{1}})$, where ${\text{P}}_{\text{t}}$ is the crude oil price at time t. Using the same LASSO-selected predictors and RMSE as the evaluation metric, the results presented in Table 21 demonstrate that our key findings remain robust. Specifically, models incorporating EUI continue to outperform those without it, and the relative advantage of RF for short-term forecasts and Attention-LSTM for long-term forecasts persists. This confirms that the predictive power of our models and the value of EUI are not artifacts of non-stationarity in price levels but reflect meaningful relationships that extend to the returns series.

**Table 21:** Forecasting performance using returns.

Models	Horizon (month)	EUI_without	EUI_equally	EUI_GDP
LR	1	31.907***	30.814***	28.683***
	3	31.066***	30.898***	30.418***
	6	58.279***	58.206***	57.648***
	12	42.242***	41.926***	41.894***
LSTM	1	31.031***	30.489***	30.024***
	3	23.112***	19.968***	21.705***
	6	25.168***	22.851***	23.982***
	12	32.745***	28.685***	30.963***
Attention-LSTM	1	24.227***	23.294***	23.758***
	3	22.300***	19.621***	21.682***
	6	23.204***	21.432***	20.293***
	12	27.132***	22.469***	27.064***
SVR	1	32.058***	30.002***	30.568***
	3	32.544***	28.187***	23.872***
	6	37.675***	36.920***	37.041***
	12	33.865***	33.544***	31.575***
RF	1	13.740***	13.237***	13.515***
	3	23.926***	22.368***	22.983***
	6	26.252***	24.879***	23.604***
	12	33.725***	28.827***	31.106***
XGBoost	1	25.444***	19.955***	22.002***
	3	26.137***	25.544***	25.046***
	6	34.342***	33.200***	33.849***
	12	32.452***	29.628***	31.737***

Note: *, **, and *** represent statistical significance at the 10%, 5%, and 1% levels, respectively.

### Different market conditions

When predicting crude oil prices, it’s critical to consider extreme events like the financial global financial crisis, the Eurozone debt crisis, and the COVID-19 pandemic because these events trigger extreme demand destruction, market panic, and policy interventions that drastically distort traditional supply-demand patterns. In this subsection, we further validate the performance of the prediction models under global financial crisis (2008–2009) and COVID-19 pandemic (2020–2023). We use the same LASSO-selected predictors and maintain the forecasting scheme with chronological splits, ensuring no future information is leaked. The evaluation metrics are also RMSE, and the prediction results are shown in Tables 22 and 23. The results show that under extreme events, the RF model with EUI still has better performance in the short term, while the Attention-LSTM model with EUI still outperforms the other prediction models in the long term, except for the 1-month-ahead XGBoost model without EUI index. We suspect that this is because XGBoost can handle nonlinear relationships and noisy data through gradient-boosted decision trees, which robustly adapt to abrupt market fluctuations and outliers. Additionally, its regularization techniques and feature importance prioritization reduce overfitting, enhancing accuracy in capturing rapid, localized price shifts during volatile periods. These results are basically consistent with previous results.

**Table 22:** Forecasting performance under global financial crisis.

Models	Horizon (month)	EUI_without	EUI_equally	EUI_GDP
LR	1	66.212***	65.339***	66.129***
	3	52.902***	50.441***	50.668***
	6	155.414***	153.028***	154.914***
	12	150.466***	149.039***	148.592***
LSTM	1	17.629***	17.326***	17.526***
	3	18.060***	17.652***	17.956***
	6	16.593***	15.874***	16.265***
	12	16.919***	16.627***	16.913***
Attention-LSTM	1	16.792***	16.476***	16.507***
	3	17.294***	17.204***	17.097***
	6	15.311***	15.124***	14.883***
	12	16.416***	15.551***	16.248***
SVR	1	25.157***	24.883***	23.132***
	3	32.978***	30.266***	30.134***
	6	31.143***	31.118***	29.314***
	12	33.121***	32.956***	31.930***
RF	1	17.215***	16.021***	16.238***
	3	25.506***	20.480***	22.183***
	6	32.346***	31.018***	28.038***
	12	26.181***	26.018***	25.638***
XGBoost	1	16.732***	16.426***	17.133***
	3	25.963***	21.887***	24.139***
	6	45.883***	40.428***	44.646***
	12	29.657***	29.547***	29.041***

Note: *, **, and *** represent statistical significance at the 10%, 5%, and 1% levels, respectively.

**Table 23:** Forecasting performance under COVID-19 pandemic.

Models	Horizon (month)	EUI_without	EUI_equally	EUI_GDP
LR	1	45.306***	44.491***	45.225***
	3	46.315***	43.939***	44.169***
	6	137.172***	134.712***	136.255***
	12	127.749***	126.059***	125.828***
LSTM	1	13.763***	12.767***	13.595***
	3	14.679***	14.094***	14.467***
	6	13.635***	12.626***	13.247***
	12	13.675***	13.623***	13.614***
Attention-LSTM	1	14.779***	14.279***	14.724***
	3	13.106***	13.030***	12.628***
	6	11.568***	11.187***	10.959***
	12	12.459***	11.702***	12.235***
SVR	1	21.338***	21.043***	19.628***
	3	28.126***	25.706***	25.910***
	6	26.739***	26.698***	25.170***
	12	28.539***	28.372***	27.487***
RF	1	13.264***	12.220***	12.669***
	3	22.226***	17.786***	19.248***
	6	27.937***	26.632***	24.369***
	12	23.438***	23.315***	23.027***
XGBoost	1	13.879***	13.720***	13.678***
	3	21.919***	18.296***	20.130***
	6	39.337***	34.414***	38.016***
	12	26.042***	25.795***	25.175***

Note: *, **, and *** represent statistical significance at the 10%, 5%, and 1% levels, respectively.

## Conclusion

This study uses the energy-related uncertainty index (EUI) to predict the crude oil prices and introduces machine learning methods to discuss how to improve the accuracy of predicting crude oil prices. Existing empirical studies regarding the impact of uncertainty on crude oil have not considered energy uncertainty and rarely use machine learning methods to assess the predictive power of uncertainty. Therefore, we explore whether the EUI and machine learning methods (i.e., LSTM, Attention-LSTM, SVR, RF, XGBoost) can help generate more accurate predictions. In this study, LR is used as the benchmark model.

Several interesting findings are highlighted here. First, the empirical results suggest that EUI is shown to have a significant impact on the crude oil prices. Second, the RF model has stronger predictive power than other competing methods in the short term, while the Attention-LSTM model exhibits better predictive performance in the long term. Additionally, our results are robust based on different feature selection method and crude oil futures. These findings will help policymakers and investors better understand the crude oil futures market and make more informed decisions about the energy market, including investment and risk management decisions.

While this paper contributes to the existing literature, there are still limitations that need to be overcome in the future. First, when analyzing the predictive capability of EUI, we comprehensively consider the financial and economic factors while overlooking the influence of political factors. The political climate all across the world is undergoing unprecedented changes, and the related factors are highly likely to impact crude oil prices in the near term, which deserves our attention. In the subsequent research, we will evaluate the predictive effectiveness of energy uncertainties after incorporating political factors and conduct an in-depth analysis of their interactive effects on crude oil prices Second, while our analysis demonstrates robustness to alternative data split, employing rolling or expanding window schemes in future work would better simulate real-time forecasting conditions. Finally, we have analyzed the crude oil market mainly based on Brent crude oil and WTI crude oil. In our future work, we will study the crude oil market more specifically, such as the Chinese crude oil market.

## Supporting information

S1 File S1_file.xls. Supported data.
(XLS)
